# Uncovering Hidden Mechanisms of Different Prescriptions Treatment for Osteoporosis *via* Novel Bioinformatics Model and Experiment Validation

**DOI:** 10.3389/fcell.2022.831894

**Published:** 2022-02-08

**Authors:** Yujie Liu, Qinwen Liu, Chuanhui Yin, Yi Li, Jie Wu, Quanlin Chen, Hailang Yu, Aiping Lu, Daogang Guan

**Affiliations:** ^1^ Department of Biochemistry and Molecular Biology, School of Basic Medical Sciences, Southern Medical University, Guangzhou, China; ^2^ Guangdong Key Laboratory of Biochip Technology, Southern Medical University, Guangzhou, China; ^3^ Department of Radiology, Nanfang Hospital, Southern Medical University, Guangzhou, China; ^4^ Institute of Integrated Bioinformedicine and Translational Science, Hong Kong Baptist University, Hong Kong SAR, China; ^5^ Guangdong-Hong Kong-Macau Joint Lab on Chinese Medicine and Immune Disease Research, Guangzhou, China

**Keywords:** osteoporosis, Gushukang Granules, Xianling Gubao Capsules, Er-xian Decoction, herbal medicine, functional response motif

## Abstract

Osteoporosis (OP) is a systemic disease susceptible to fracture due to the decline of bone mineral density and bone mass, the destruction of bone tissue microstructure, and increased bone fragility. At present, the treatments of OP mainly include bisphosphonates, hormone therapy, and RANKL antibody therapy. However, these treatments have observable side effects and cannot fundamentally improve bone metabolism. Currently, the prescription of herbal medicine and their derived proprietary Chinese medicines are playing increasingly important roles in the treatment of OP due to their significant curative effects and few side effects. Among these prescriptions, Gushukang Granules (GSK), Xianling Gubao Capsules (XLGB), and Er-xian Decoction (EXD) are widely employed at the clinic on therapy of OP, which also is in line with the compatibility principle of “different treatments for the same disease” in herbal medicine. However, at present, the functional interpretation of “different treatments for the same disease” in herbal medicine still lacks systematic quantitative research, especially on the detection of key component groups and mechanisms. To solve this problem, we designed a new bioinformatics model based on random walk, optimized programming, and information gain to analyze the components and targets to figure out the Functional Response Motifs (FRMs) of different prescriptions for the therapy of OP. The distribution of high relevance score, the number of reported evidence, and coverage of enriched pathways were performed to verify the precision and reliability of FRMs. At the same time, the information gain and target influence of each component was calculated, and the key component groups in all FRMs of each prescription were screened to speculate the potential action mode of different prescriptions on the same disease. Results show that the relevance score and the number of reported evidence of high reliable genes in FRMs were higher than those of the pathogenic genes of OP. Furthermore, the gene enrichment pathways in FRMs could cover 79.6, 81, and 79.5% of the gene enrichment pathways in the component-target (C-T) network. Functional pathway enrichment analysis showed that GSK, XLGB, and EXD all treat OP through osteoclast differentiation (hsa04380), calcium signaling pathway (hsa04020), MAPK signaling pathway (hsa04010), and PI3K-Akt signaling pathway (hsa04151). Combined with experiments, the key component groups and the mechanism of “different treatments for the same disease” in the three prescriptions and proprietary Chinese medicines were verified. This study provides methodological references for the optimization and mechanism speculation of Chinese medicine prescriptions and proprietary Chinese medicines.

## Introduction

Osteoporosis (OP) is the most common bone disease characterized by decreased bone mass and degradation of bone microstructure. The main clinical manifestations are decreased bone density, chronic pain, decreased mobility, and so on (Miller, 2016). The common pathogenic factors include aging, decreased estrogen, nutritional disorders, poor living habits, and long-term use of steroids, anti-cancer drugs, diuretics, and so on. The main manifestations of OP are the decline of bone mineral density and bone quality. Its symptoms are most prone to systemic metabolic diseases such as fracture, low back pain, shortening of body length, bone pain, and even weakening of respiratory function (Zhao and Wang, 2003). It has a significant influence on the quality of life of patients and brings a heavy economic burden to families and society. OP is universal and can affect men and women of all races, especially older women who have passed menopause (Lane et al., 2000).

The current treatment drugs for OP mainly include estrogen (Stefanick, 2005), bisphosphonates (Delmas, 2005), calcitonin (Silverman, 2003), and parathyroid hormone (Neer et al., 2001; Srivastava and Deal, 2002). After treatment, the symptoms of osteoporosis patients will be alleviated, but these treatments cannot radically enhance bone metabolism and maintain the balance between osteoclasts and osteogenesis. In addition, the side effects of drugs also bring multiple risks to patients, including some side effects, even toxicity to the kidney, blood, and liver; gastrointestinal side effects; and immunosuppression (Tella and Gallagher, 2014). After taking estrogen receptor modulator drugs, it can also cause endometrial hyperplasia and uterine bleeding (Papapoulos and Makras, 2008).

Herbal medicine has been diffusely used at the clinic on OP therapy due to its fewer side effects and irritation (Zhang et al., 2016). It is known that Gushukang Granules (GSK), Xianling Gubao Capsules (XLGB), Er-xian Decoction (EXD), Liuwei Dihuang Pills, and Guishen Pill are effective in treating OP. In the treatments of OP, different prescriptions have the same and different targets and pathways, which fully figure out the action mode of “multi-components-multi-targets-multi-pathways” on the clinical therapy of complex diseases. How to systematically quantify the hidden mode of action in herbal prescriptions is the foundation and crux to interpret the principle of “different treatments for the same disease” in herbal medicine.

Among these prescriptions, GSK, XLGB, and EXD are widely used in the clinic. GSK contains seven herbs: *Epimedium brevicornum* Maxim., *Radix Rehmanniae Preparata*, *Auricularia auricular* (L.) Underw., *Astragalus mongholicus* Bunge, *Cucumis sativus* L., *Davallia mariesii* Moore ex Bak., and *Salvia miltiorrhiza* Bge. XLGB consists of six herbs: *Epimedium brevicornum* Maxim., *Rehmannia glutinosa* (Gaetn.) Libosch. ex Fisch. et Mey*.*, *Salvia miltiorrhiza* Bge*.*, *Dipsacales*, *Anemarrhena asphodeloides* Bunge, and *Psoralea corylifolia* Linn. EXD has six kinds of herbs: *Curculigo orchioides* Gaertn., *Epimedium brevicornum* Maxim., *Angelica sinensis* (Oliv.) Diels, *Morinda officinalis* How., *Phellodendron chinense* Schneid., and *Anemarrhena asphodeloides* Bunge. Clinical studies have shown that GSK can increase sex hormones (estrogen and androgen), inhibit bone absorption, effectively improve bone mineral density (BMD), reduce bone loss, increase osteocalcin (OC) and blood alkaline phosphatase (ALP) levels, and enhance osteoblast activity, which can effectively prevent and treat OP (Li et al., 2001; Wang et al., 2007; Wang et al., 2018a). Pharmacological research has shown that XLGB can improve bone metabolism, promote osteogenic effects, inhibit osteoclasts, increase bone density, and facilitate bone formation (Zhu and Hou, 2020). Clinical studies have also shown that XLGB has a therapeutic effect on BMD in patients with OP, which can effectively increase BMD, improve bone metabolism, and control bone loss (Wu et al., 2017). Pharmacological studies have shown that EXD not only increases the proliferation of osteoblasts and alkaline phosphatase (ALP) activity but also reduces the tartrate resistant acid phosphatase (TRAP) activity of osteoclasts (Li et al., 2017). Additionally, EXD affects the calcium signaling pathway and mediates apoptosis by activating the expression of downstream CAMK and activates downstream JNK, AKT, and ERK through upstream TNF-α, affecting the apoptosis process of bone-related cells (Yang et al., 2021). However, the pharmacodynamic material foundation and related molecular mechanism of different prescriptions in the therapy of OP under the concept of “different treatments for same disease” are still indistinct. Therefore, it is necessary to scientifically decipher the material basis and molecular mechanism of the efficacy of different prescriptions on the same disease.

Herbal informatics is an interdisciplinary subject that integrates Chinese medicine, computer science, biology, mathematics, multi-directional pharmacology, and other disciplines. It researches complex herbal medicine systems by systematically observing the response and effect of drugs on pathogenetic gene networks (Wang et al., 2021). Herbal informatics and network pharmacology have been diffusely used in the “same disease and different treatments” of herbal medicine. For example, Wang Kexin et al. clarified the molecular mechanism of DSD, GFD, and HGWD in treating rheumatoid arthritis based on herbal informatics (Wang et al., 2020a). Gao Yao et al. used the method of herbal informatics to analyze the mechanism of Xiaoyao Powder and Kaixin powder in the treatment of depression (Gao et al., 2018). Zhao Can et al. discussed the possible mechanism of Fuling Xingren Gancao Decoction and Ju-Zhijiang Decoction in treating coronary atherosclerotic heart disease based on the herbal informatics method (Zhao et al., 2019). With the continuous in-depth intersection of life sciences, chemistry, computer and information sciences, and other disciplines with drug research and the optimization and upgrading of network visualization tools and network construction analysis technologies, the research ideas and technical methods of herbal informatics will be better used. The study of the mechanism of the “different treatments for the same disease” of Chinese medicine provides more reference for the study of modernization of Chinese medicine (Li and Zhang, 2013).

In this study, a computational model based on herbal informatics was designed to discover the Significant Different Functional Modes (SDFMs) of different prescriptions in the therapy of OP. In order to further decode the key components group of different prescriptions on OP, a genetic algorithm-based optimization model was designed to figure out the FRMs from SDFMs. The distribution of high relevance score, the number of reported evidence, and the coverage of the enrichment pathway of target genes in FRMs were used to evaluate the accuracy and reliability of the FRMs detection model. Then, the effective proteins in the FRMs of each prescription were employed to screen the key components group of each prescription. The importance scores of the components in the key components group of each prescription were obtained based on information gain and target influence, and the effects of these key components were verified by cell experiments. Finally, the potential action mode of different prescriptions in treating the same disease was speculated based on the key components group of each prescription.

In conclusion, according to the herbal informatics strategy proposed in this study, the main mechanisms and relevant pharmacological effects of different treatments of OP can be detected through FRMs, which provide a new network-based method for herbal medicine in the clinic therapy of complex diseases.

## Materials and Methods

### Flowchart

**FIGURE 1:**
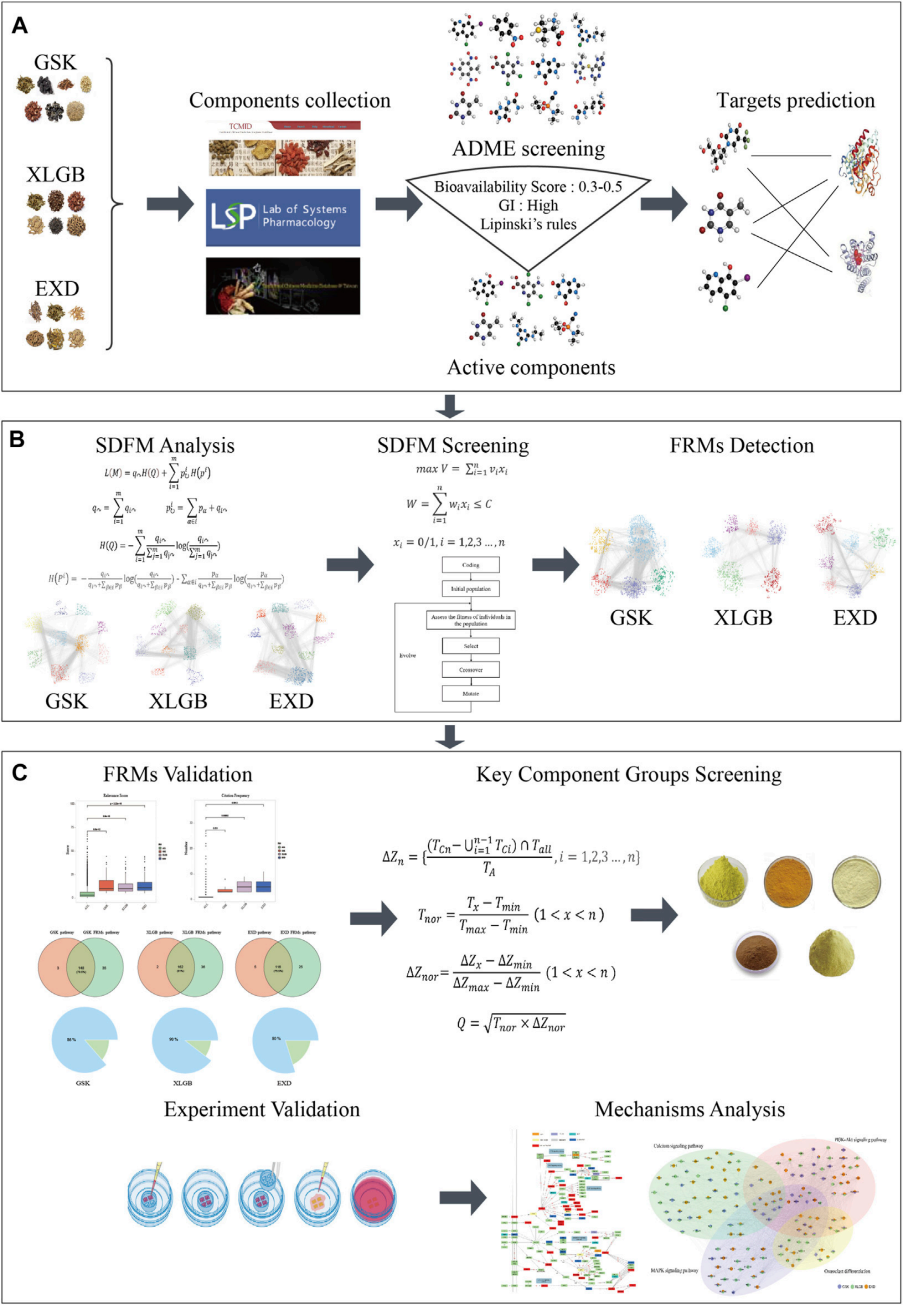
The flowchart of our network pharmacology approach, including component collection and target prediction **(A)**. Using the function motif discovery model to find Significant Different Functional Modes (SDFMs) in the three prescriptions and then using the genetic-based optimization model to figure out the Functional Response Motifs (FRMs) **(B)**. Validation of FRMs, screening key component groups, and experiment validation and potential mechanism analysis **(C)**.

The schematic diagram of the whole process is shown in Figure 1, and the detailed procedure is described as follows: 1) chemical compounds of GSK, XLGB, and EXD were extracted from the published databases, and a widely used ADME screening model was used to screen potential active components in these compounds. Then, the online webserver is used to predict the targets of these active components. 2) The discovery model of the functional response motif was designed to optimize the C-T network and obtain Significant SDFMs. SDFMs were optimized by a genetic-based optimization model to obtain FRMs. 3) The reliability and accuracy of the FRMs detection model were validated by the distribution of the relevance score, the number of reported evidence, and coverage of functional pathways. 4) The information gain and target influence were combined to score the components in the key components group of each prescription. 5) The effectiveness of high-scored and randomly selected components in the key components group were performed in the *in vitro* experiments to confirm the precision of our proposed key components group selection model. 6) Finally, the potential action mode of different prescriptions treating the same disease mechanism was inferred by function analysis of the key components group in each prescription.

### Component Collection

The original components in the three prescriptions were extracted by searching the Traditional Chinese Medicine Integrated Database (TCMID 2.0), TCM Database@Taiwan, and Traditional Chinese Medicine Systems Pharmacology Database and Analysis Platform (TCMSP) with the herbs in GSK, XLGB, and EXD as keywords. Open Babel (version 2.4.1) was used to convert the structures of all chemical components into canonical SMILES. The Similarity Ensemble Approach (SEA), Hit Identification and Target Prediction (HitPick), and SwissTargetPrediction were used to predict the drug targets of GSK, XLGB, and EXD.

### SwissADME Screening

Lipinski’s Rule of Five is the five elementary principles for selecting drug-like molecules, put forward by the pharmaceutical chemist Christopher Lipinski in 1997. Compounds that conform to the Lipinski Rule of Five will have better pharmacokinetic features and higher bioavailability during metabolism *in vivo*, so they tend more to become oral drugs. In drug research and development, the Lipinski Rule of Five is used in the preliminary screening of the compound library to exclude those molecules that are unfit for drugs, narrow the range of selecting, and economize the cost of drug research and development. The detailed description of Lipinski’s Rule of Five is that the molecular weight of the compound is less than 500 Da. The number of hydrogen bond donors (including hydroxyl groups and amino groups) in the structure of the compound does not exceed 5. The number of hydrogen bond receptors in the compound does not exceed 10. The logarithmic value (logP) of the lipid-water partition coefficient of the compound is between −2 and 5. The quantity of rotatable bonds in the compound does not exceed 10 (Lipinski et al., 2001). The bioavailability score indicates the probability that a compound has at least 10% oral bioavailability or a measurable Caco-2 permeability in rats. Gastrointestinal absorption (GI absorption) indicates that the drug has good oral bioavailability (Daina et al., 2017). Our research screened the active components of GSK, XLGB, and EXD according to Lipinski’s Rule of Five, bioavailability, and gastrointestinal absorption. Among them, the bioavailability is defined between 0.3 and 0.55.

### Network Construction

Cytoscape (version 3.7.2) was employed to build a C-T network, and its plug-in NetworkAnalyzer was used to analyze network topology parameters.

### Functional Enrichment Analysis

Function enrichment is conducted based on KEGG (Kyoto Encyclopedia of Genes and Genomes). The hypergeometric distribution model is employed to calculate the significance of the biological pathway containing the target gene. The Benjamini–Hochberg method was used to correct the *p*-value. All statistical analysis was performed using the R language (version 4.0.5).

### Explore the Significant Different Functional Modes

In order to find SDFMs in the three prescriptions for treating OP, we designed the below function motif discovery model.

The function motif discovery model is based on the combination of random walk and information compression. It uses the double-layer Huffman coding method to associate community discovery with information coding, records the paths generated by a random walk in the graph, and finds the community division with the shortest length. The average coding length of each step described in the random walk can be measured by the following prescription:

$$L\left(M\right)=q_{↷}H\left(Q\right)+\overset{m}{\underset{i=1}{\sum }}p_{↻}^{i}H\left(p^{i}\right),$$

where M indicates the way of community division and  M indicates that nodes are divided into M communities.

$q_{↷}=\;\sum_{i=1}^{m}q_{i↷}$ means the proportion of all codes representing the community’s name in the code, and $q_{↷}$ is equal to the probability of jumping out of community i .

$H\left(Q\right)=\;-\sum_{i=1}^{m}\frac{q_{i↷}}{\sum_{j=1}^{m}q_{j↷}}\log\left(\frac{q_{i↷}}{\sum_{j=1}^{m}q_{j↷}}\right)$ stands for the average length of bytes required to encode a community name.

$p_{↻}^{i}=\sum_{\alpha \in i}p_{\alpha }+q_{i↷}$ shows the code proportion of all nodes (including jump nodes) belonging to community i in the code.

$H\left(P^{i}\right)$ denotes the average byte length required by all nodes in the coding community i . The average coding length ***L*** (***M***) of each step is the weighted sum of two parts. One part is the average byte length required by the coding community name, and the other part is the average byte length required by the coding node in each community. The prescription can be expressed as

$$H\left(P^{i}\right)=\;-\frac{q_{i↷}}{q_{i↷}+\sum_{\beta \in i}p_{\beta }}\log\left(\frac{q_{i↷}}{q_{i↷}+\sum_{\beta \in i}p_{\beta }}\right)-\sum_{\alpha \in i}\frac{p_{\alpha }}{q_{i↷}+\sum_{\beta \in i}p_{\beta }}\log\left(\frac{p_{\alpha }}{q_{i↷}+\sum_{\beta \in i}p_{\beta }}\right),$$

### Detection of Functional Response Motifs

In order to further screen the SDFM, we designed a novel genetic-based optimization model, which is described in detail as follows:

$$\mathrm{max V}=\;\overset{n}{\underset{i=1}{\sum }}v_{i}x_{i},$$

$$W=\overset{n}{\underset{i=1}{\sum }}w_{i}x_{i}\leq C,$$

$$x_{i}=0/1,i=1,2,3\ldots ,n,$$

The genetic algorithm (GA) is a random walk method that simulates the evolution of the genetic mechanism of the evolutionary laws of nature. It takes all individuals in a population as the object and efficiently searches a coded parameter space through genetic operations of selection, crossover, and mutation. As a new global optimization search algorithm, the genetic algorithm has the obvious characteristics of simplicity and generality, strong robustness, high efficiency, and practicality. It can be applied in all kinds of fields, has achieved good results, and has become one of the critical, intelligent algorithms by degrees. The calculation process is as follows:

1) Random generation of the initial population: the chromosome coding method is represented by a binary column code of length n . When $x_{i}=0$ , the binary code is 0. Otherwise, the binary code is 1. A binary column is a chromosome.
2) Individual evaluation: under the premise of not exceeding C , $v_{i}$ and $\overset{n}{\underset{i=1}{\sum }}v_{i}x_{i}$ were used to evaluate individual fitness.
3) Individual selection: the roulette model is used to convert individual fitness into the area of the roulette wheel in proportion and rotate the roulette wheel. Finally, the individual corresponding to the landing position is selected.
4) Two points cross: two points are randomly set in the individual code, and some genes are exchanged in the middle of the two intersection points.
5) Basic mutation, a number is randomly generated for each chromosome, indicating whether the chromosome needs to be mutated. If a mutation is needed, a random variable is generated, indicating which bit of the chromosome to modify.
6) A new population is formed, and the iteration continues until the termination condition is met: W=C .

### Pathway Network Integrating

Cytoscape (version 3.7.2) was used to combine osteoclast differentiation, calcium signaling pathway, MAPK signaling pathway, and PI3K-Akt signaling pathway into an integrated pathway.

### Key Component Groups Screening

In order to screen the key component groups in different prescriptions, we designed a novel component importance calculation method that combined the information gain and target influence. We sort all the components in descending order of the corresponding target number and calculate the information gain Z of each component. The degree of information gain represents the contribution of increased coverage to the whole targets after adding the component. Higher information gain score indicates the influence and the importance of the component in the C-T network of each prescription. There are n components, the corresponding target set of the components is denoted by $T_{\mathrm{Ci}}$ , the target set of all FRMs is denoted by $T_{\mathrm{all}}$ , and the target number of all FRMs is denoted by $T_{A}$ . The information gain Z of components is calculated according to the following prescription. Then, we standardize the target number T of the component and the information gain Z and calculate the Q score. That is,

$$\Delta Z_{n}=\left\{\frac{\left(T_{\mathrm{Cn}}-\cup_{i=1}^{n-1}T_{\mathrm{Ci}}\right)\cap T_{\mathrm{all}}}{T_{A}},i=1,2,3\ldots ,n\right\},$$

$$T_{\mathrm{nor}}=\frac{T_{x}-T_{\min}}{T_{\max}-T_{\min}}\;\left(1<x<n\right),$$

$$\Delta Z_{\mathrm{nor}}=\frac{\Delta Z_{x}-\Delta Z_{\min}}{\Delta Z_{\max}-\Delta Z_{\min}}\;\left(1<x<n\right),$$

$$Q=\sqrt{T_{\mathrm{nor}}\times \Delta Z_{\mathrm{nor}}}.$$

### Experiment Validation

#### Cell Culture and Drug Treatment

Mouse preosteoblastic MC3T3-E1 cells were purchased from the American Type Culture Collection (ATCC) and stored in Minimal Essential Medium, Alpha (α-MEM), supplemented with 10% fetal bovine serum (FBS), 100 units/mL of penicillin G, and 100 μg/ml of streptomycin at 37°C under 5% CO_2_. Cells were seeded into 96-well plates (1×10^3^ cells per/well) for 24 h and then treated with 5 μM quercetin, isoliquiritigenin, rutaecarpine, isofraxidin, and secoisolariciresinol for 24 and 48 h. Quercetin, isoliquiritigenin, rutaecarpine, isofraxidin, and secoisolariciresinol (≥98% purity by HPLC) were dissolved in DMSO.

#### Cell Viability Assay

Cell Count Kit-8 (CCK-8) assay was utilized to measure cell viability. After cell culture, add 10 μl CCK8 to the culture medium and incubate at 37°C for 2 h. The absorbance was measured at 450 nm with a microplate reader (*TECAN, infiniteM200*).

## Results

### Collection of Chemical Components and Determination of High-Concentration Components

We collected the herbal components and concentrations of GSK, XLGB, and EXD from the reported literature. The detailed information is shown in Table 1 and Supplementary Table S1. The results show that the chemical composition and concentration of the herbal medicine provide experimental auxiliary evidence for searching for active components and provide a valuable reference for further analysis.

**TABLE 1:** The experiments confirmed high concentration components of GSK, XLGB, and EXD.

Herb	Method	Component	Concentration (mg/g)	Prescription	References
*Epimedium brevicornum *Maxim.	HPLC	Icariin	2.025	GSK	SZ Sun, HE Yan-Li, YW Wen, & YT Chen. (2019). Simultaneous determination of six components in Gushukang Granula and Gushukang Capsules by HPLC-ms/ms. Chinese Journal of Pharmaceutical Analysis
*Radix Rehmanniae Preparata*		Acteoside	0.043		
*Astragalus mongholicus *Bunge		Calycosin-7-glucoside	0.06		
*Salvia miltiorrhiza* Bge.		Tanshinone ⅡA	0.0858		
*Davallia mariesii *Moore ex Bak		Naringin	0.1049		
*Epimedium brevicornum *Maxim	HPLC	Icariin	2.119	GSK	YE Guangming, Y. Jiang, Y. Chen, GU Liping, & X. Xue. (2010). Simultaneous determination of contents of icariin and naringin in Gushukang Granules by HPLC method. Pharmaceutical Care and Research
*Davallia mariesii *Moore ex Bak.		Naringin	0.163		
*Epimedium brevicornum *Maxim.	HPLC	Icariin	1.164	XLGB	Chen, Z., Xiaoxia, L., Chen, G., & Chen, J. (2017). One-step assay for five components in Xianling Gubao Capsule by HPLC method. Journal of Pharmaceutical Practice
		Epimedin C	7.068		
*Dipsacales*		Asperosaponin Ⅵ	8.458		
*Psoralea corylifolia* Linn.		Psoralen	0.776		
		Angelicin	0.838		
*Dipsacales*	HPLC	Asperosaponin Ⅵ	8.458	XLGB	Gong, QD, Chen, ZL, and Chen, G. Q. (2016). Determination of asperosaponin Ⅵ, psoralen, and angelicin in Xianling Gubao Capsule by HPLC. Chinese Traditional and Herbal Drugs
*Psoralea corylifolia* Linn.		Psoralen	0.776		
		Angelicin	0.838		
*Epimedium brevicornum *Maxim.	HPLC	Epimedin B	0.89	EXD	Gao, F., Liu, Y., H Li, Fan, F., & Pharmacy, D. O. (2019). Determination of epimedium flavonoids in Er-xian Decoction by high-performance liquid chromatography. World Chinese Medicine
		Epimedin C	0.701		
		Icariin	0.487		
		Icariside II	1.027		

### Screening of Active Components for GSK, XLGB, and EXD

**FIGURE 2:**
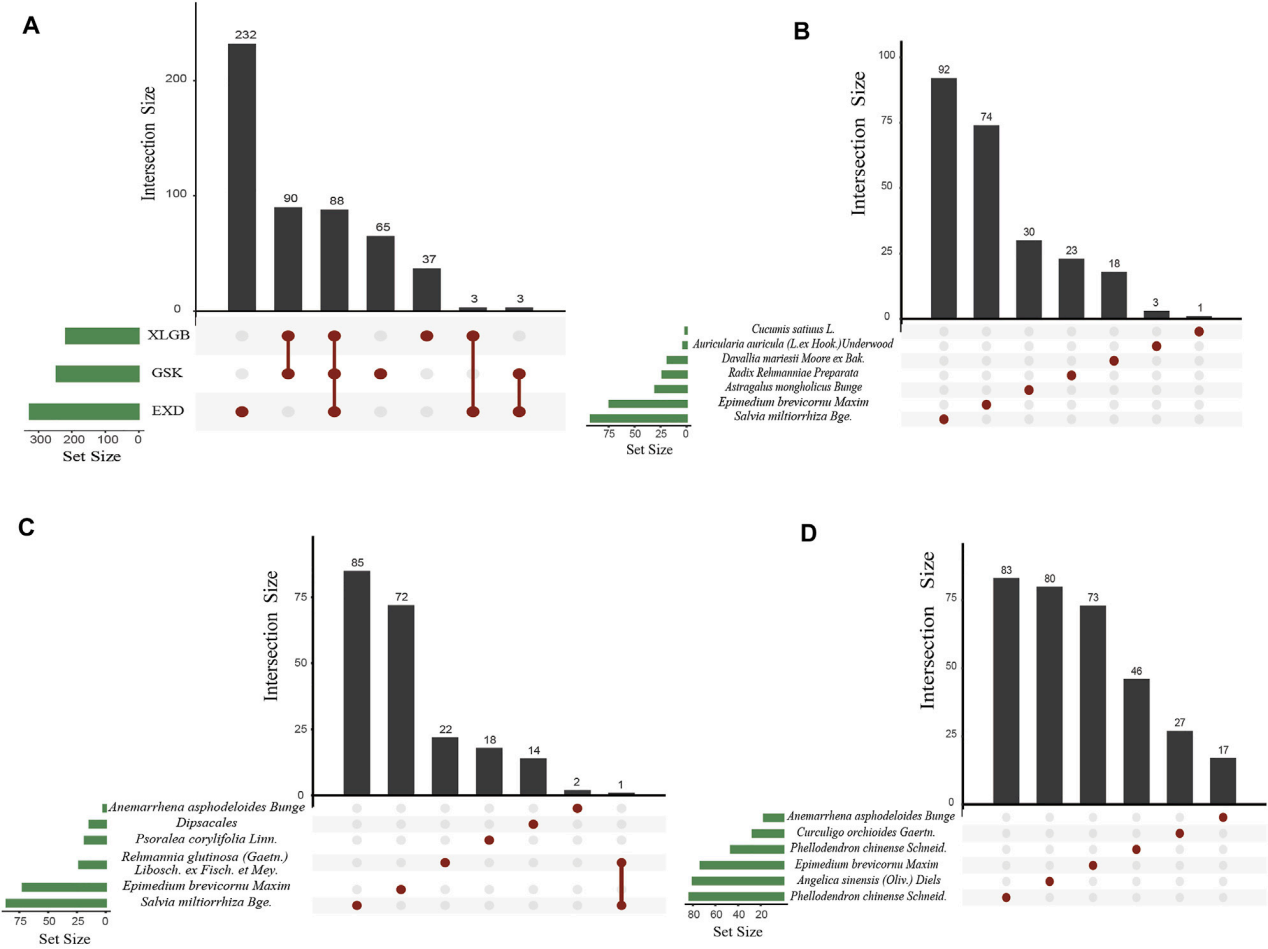
Distribution map of active components in GSK, XLGB, and EXD **(A)**. Common components of GSK, XLGB, and EXD **(B–D)**. Distribution map of herbs in GSK **(B)**. XLGB **(C)**. and EXD **(D)**.

Seven herbs of GSK with 672 components, six herbs of XLGB with 540 components, and six herbs of EXD with 752 components were extracted from the Traditional Chinese Medicine Integrated Database (TCMID 2.0), TCM@TAIWAN, and Traditional Chinese Medicine Systems Pharmacology Database and Analysis Platform (TCMSP). Generally speaking, each Chinese medicine compound contains multiple herbal medicines, and each herbal medicine contains a series of chemical components. The pharmacological properties of these components are closely related to their therapeutic effects. Ingredients with better pharmacological properties may have positive therapeutic effects. Therefore, before analyzing the pharmacological effects, we first screen for pharmacological properties. SwissADME was used to screen the components in accordance with Lipinski’s Rule of Five, gastrointestinal absorption, and bioavailability. After SwissADME screening, 271, 242, and 344 active components were obtained in GSK, XLGB, and EXD, respectively (Table 2, Supplementary Table S2). Our further analysis revealed 88 common components in the three prescriptions, 65, 37, and 232 specific components in GSK, XLGB, and EXD, respectively. In addition, we found that each of the herbs from GSK, XLGB, and EXD had its own chemical composition. There were many common components among the three prescriptions, while there were few common components in the prescriptions. Each prescription depended on its unique components (Figure 2). These results indicated that three prescriptions may play a role in treating OP by influencing both the common and specific components.

**TABLE 2:** The number of active components before and after SwissADME screening in GSK, XLGB, and EXD.

Formula	Chemical composition quantity before SwissADME screening	Chemical composition quantity after SwissADME screening
GSK	672	271
XLGB	540	242
EXD	752	344

### Target Prediction and C-T Network Construction

Cytoscape was used to construct a C-T network to analyze the relationships between the active components and targets of the three prescriptions (Supplementary Figure S1). The results showed 271, 240, and 339 active components; 1,264, 1,157, and 1,445 targets; and 9,701, 7,001, and 10,333 interactions in the GSK network, XLGB network, and EXD network, respectively. Then, we use the Cytoscape plug-in tool NetworkAnalyzer to further analyze the topological parameters of the three prescription C-T networks. After analyzing the network topology in the C-T network of GSK, the average degree of the components was 35.80. Among them, phenylalanine had the highest node degree, acting on 207 drug targets (degree: 207), and tyrosine acted on 128 targets in descending order (degree: 128). Quercetin acted on 120 targets (degree: 120). Studies have shown that high phenylalanine levels can affect bones, cause bone-related diseases, and affect bone mineral density (BMD) in a lower way (Mendes et al., 2012). Phosphorylation of tyrosine residues is key to the regulation of osteoclast production and bone resorption activity (Shalev and Elson, 2019). Phenylalanine can be converted to tyrosine, and the metabolic state of phenylalanine is related to normal body growth and maintenance of normal physiological functions (Westbroek et al., 2001). In addition, phenylalanine interacts with the calcium-sensing receptor (CaSR), affecting the body’s calcium metabolism and bone balance (Conigrave et al., 2008). Animal experiments have proved that OP can be prevented by intervening phenylalanine metabolism in rats (Liu et al., 2012). Quercetin can restrain the expression of ERK1/2, MAPK mRNA, and protein, thereby inhibiting the conduction of the ERK1/2-MAPK signaling pathway, promoting the expression of specific genes in bone and osteoblast generation, increasing bone mineral density, and preventing OP (Casado-Díaz et al., 2016). In the C-T network of GSK, the average degree of the target was 7.67. Among them, the highest degree of the node was Microtubule Associated Protein Tau (MAPT), which targeted 172 compounds (degree: 172), followed by Tyrosyl-DNA phosphodiesterase 1 (TDP1), which targeted 135 compounds (degree: 135). Muscleblind Like Splicing Regulator 1 (MBNL1) targeted 83 compounds (degree: 83).

In the C-T network of XLGB, the average degree of the components was 30.48. Among them, phenylalanine had the highest node degree, acting on 202 drug targets (degree: 202), and tyrosine acted on 124 targets in descending order (degree: 124). Quercetin acted on 106 targets (degree: 106). The average degree of the target was 6.05. Among them, the highest degree of the node was Microtubule Associated Protein Tau (MAPT), which targeted 105 compounds (degree: 105), followed by Tyrosyl-DNA phosphodiesterase 1 (TDP1), which targeted 74 compounds (degree: 74). Carbonic anhydrase 7 (CA7) targeted 63 compounds (degree: 63).

In the C-T network of EXD, the average degree of the components was 30.58. Among them, asperglaucide had the highest node degree, acting on 267 drug targets (degree: 267), and phenylalanine acted on 197 targets in descending order (degree: 197). n-cis-Feruloyltyramine acted on 177 targets (degree: 177). The average degree of the target was 7.16. Among them, the highest degree of the node was cytochrome P450 family 1 subfamily B member 1 (CYP1B1), which targeted 94 compounds (degree: 94), followed by carbonic anhydrase 3 (CA3), which targeted 90 compounds (degree: 90). Carbonic anhydrase 7 (CA7) targeted 86 compounds (degree: 86). Through the comparative analysis of the number of targeted genes, it can be seen that GSK had a stronger control rate on the C-T network.

The results showed that, in the three prescriptions, there were a relationship between one component corresponding to multiple targets and a phenomenon of different components acting on the same target, consistent with the function of “multi-components-multi-targets-multi-pathways” in herbal medicine, reflecting the complicacy of the underlying mechanism of herbal medicine.

### Screening Significant Different Functional Modes and Functional Response Motifs

**FIGURE 3:**
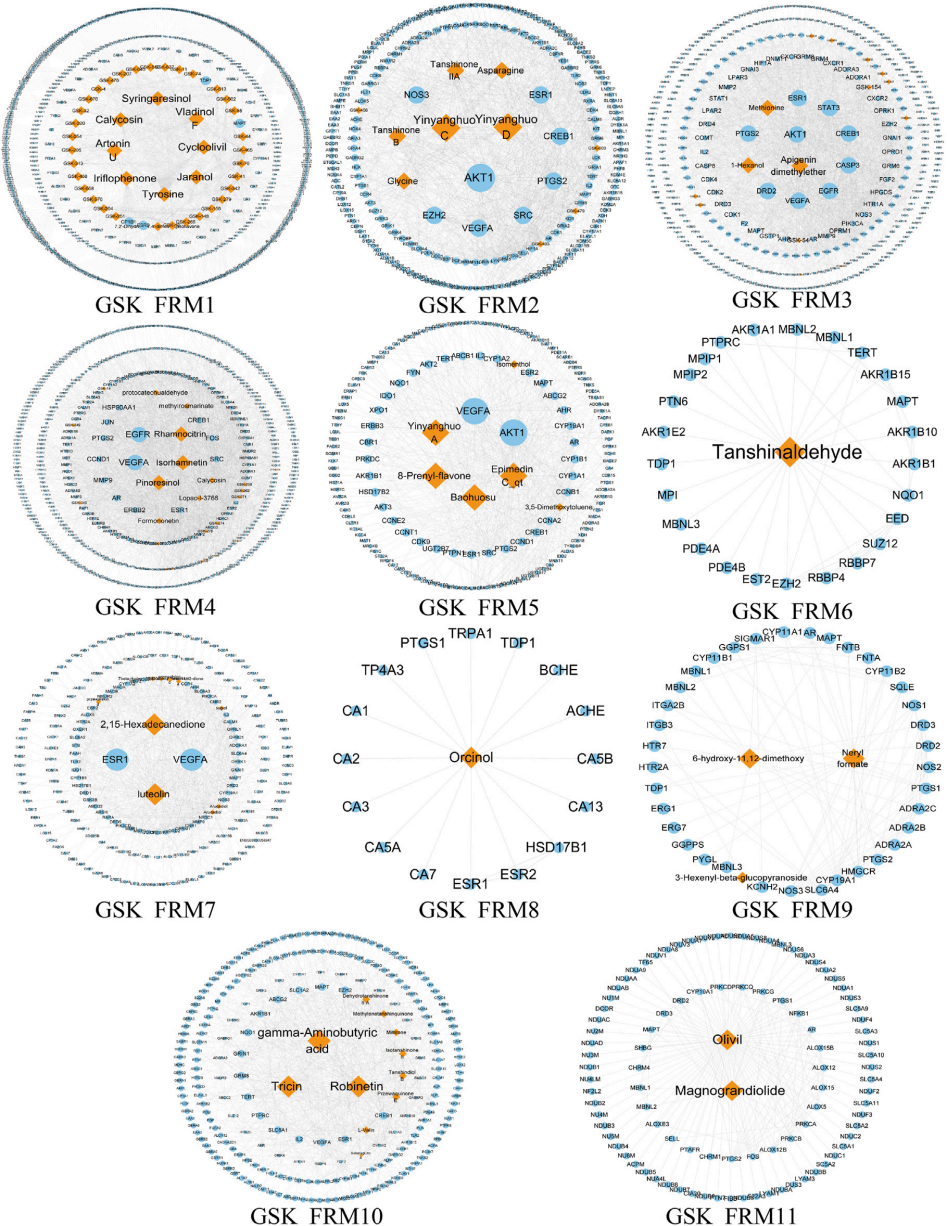
The predicated FRMs of the C-T network in GSK. The orange nodes represent the components in GSK, and the blue nodes represent the related targets.

**FIGURE 4:**
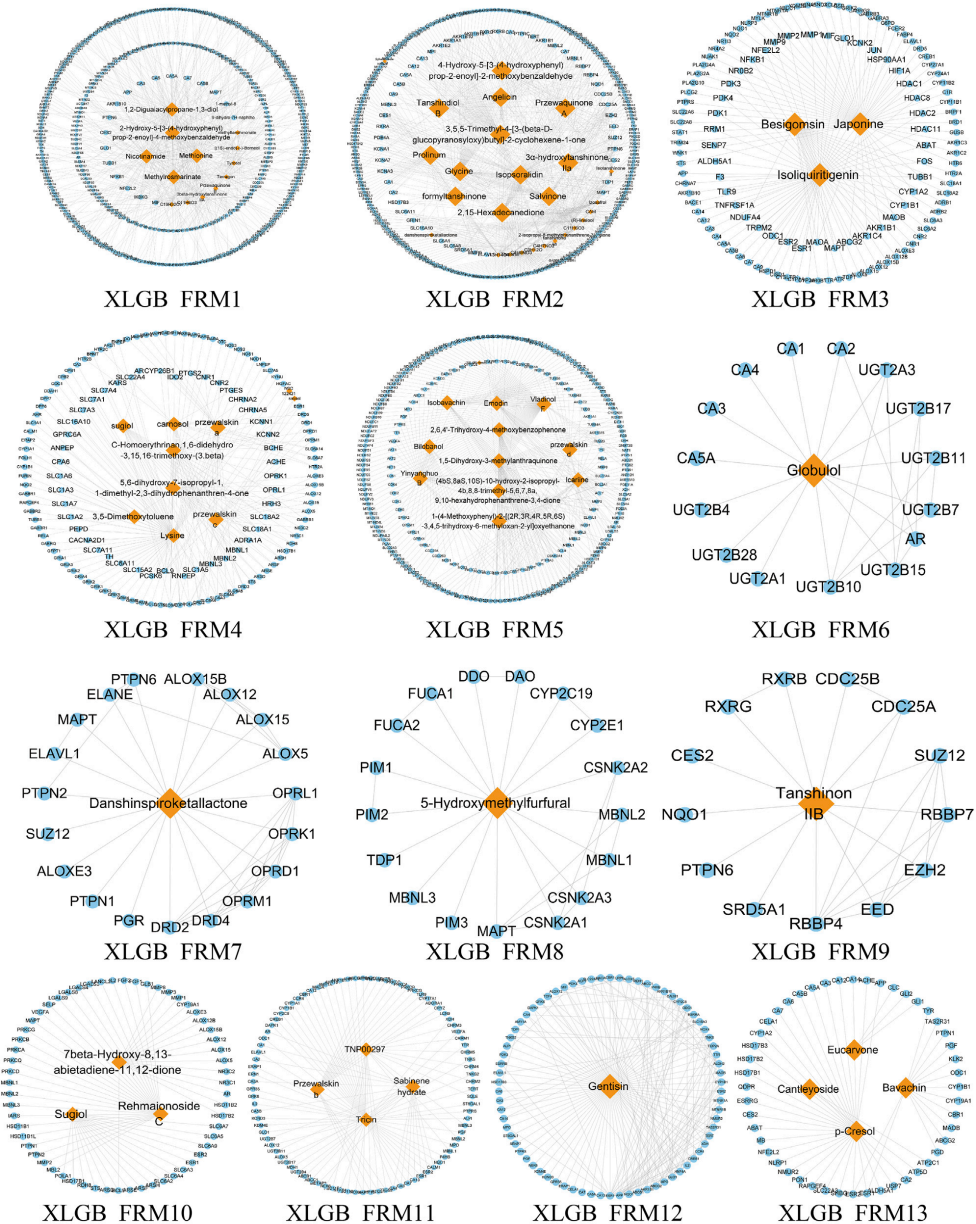
The predicated FRMs of the C-T network in XLGB. The orange nodes represent the components in XLGB, and the blue nodes represent the related targets.

**FIGURE 5:**
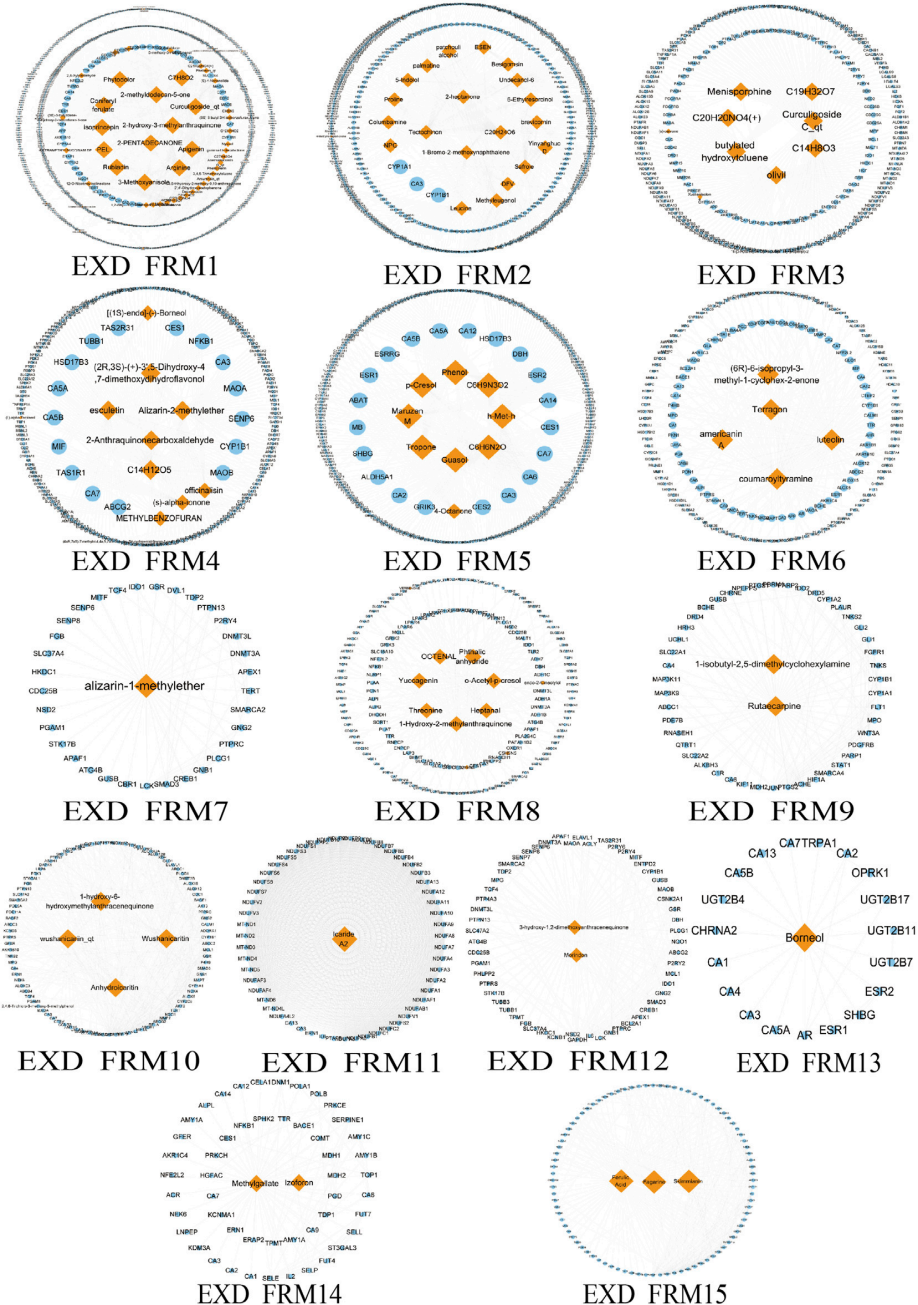
The predicated FRMs of the C-T network in EXD. The orange nodes represent the components in EXD, and the blue nodes represent the related targets.

Due to the large and complex C-T network of the three prescriptions, it is difficult to quickly extract the most important active ingredient information. Therefore, we designed a novel function motif discovery model to screen the C-T network to filter the modes that can represent the complete C-T network. The results showed that we predicted 22, 23, and 30 SDFM in GSK, XLGB, and EXD, respectively (*p* < 0.05). In order to figure out the FRMs and remove the noise in the SDFM in each prescription. We designed another genetic-based optimization model to optimize the SDFM and finally obtained 11, 13, and 15 FRMs in GSK, XLGB, and EXD, respectively (Figures 3–5).

### Validating FRMs

In order to verify whether the entire C-T network can be replaced by the predicted FRMs, we propose two criteria to evaluate the accuracy and dependability of FRMs. The first criterion is whether the relevance score and the number of reported evidence of FRMs-related high reliable genes, defined in the following section, are higher than those of the pathogenic genes of OP. High relevance score and the high number of reported evidence indicate that the genes in FRMs can cover the high reliable pathogenic genes to the maximum extent. The second criterion is whether the gene enrichment pathways in FRMs can cover the gene enrichment pathways in the C-T network as much as possible. The high pathway coverage indicates that FRMs can cover most of the gene enrichment pathways in the C-T network and have the most likely function of the prescription. Details of the results are as follows:

#### Validating FRMs Based Relevance Score and the Number of Reported Evidence

We extracted the pathogenic genes of OP with relevance score and the number of reported evidence from GeneCards database and DisGeNet database.

**FIGURE 6:**
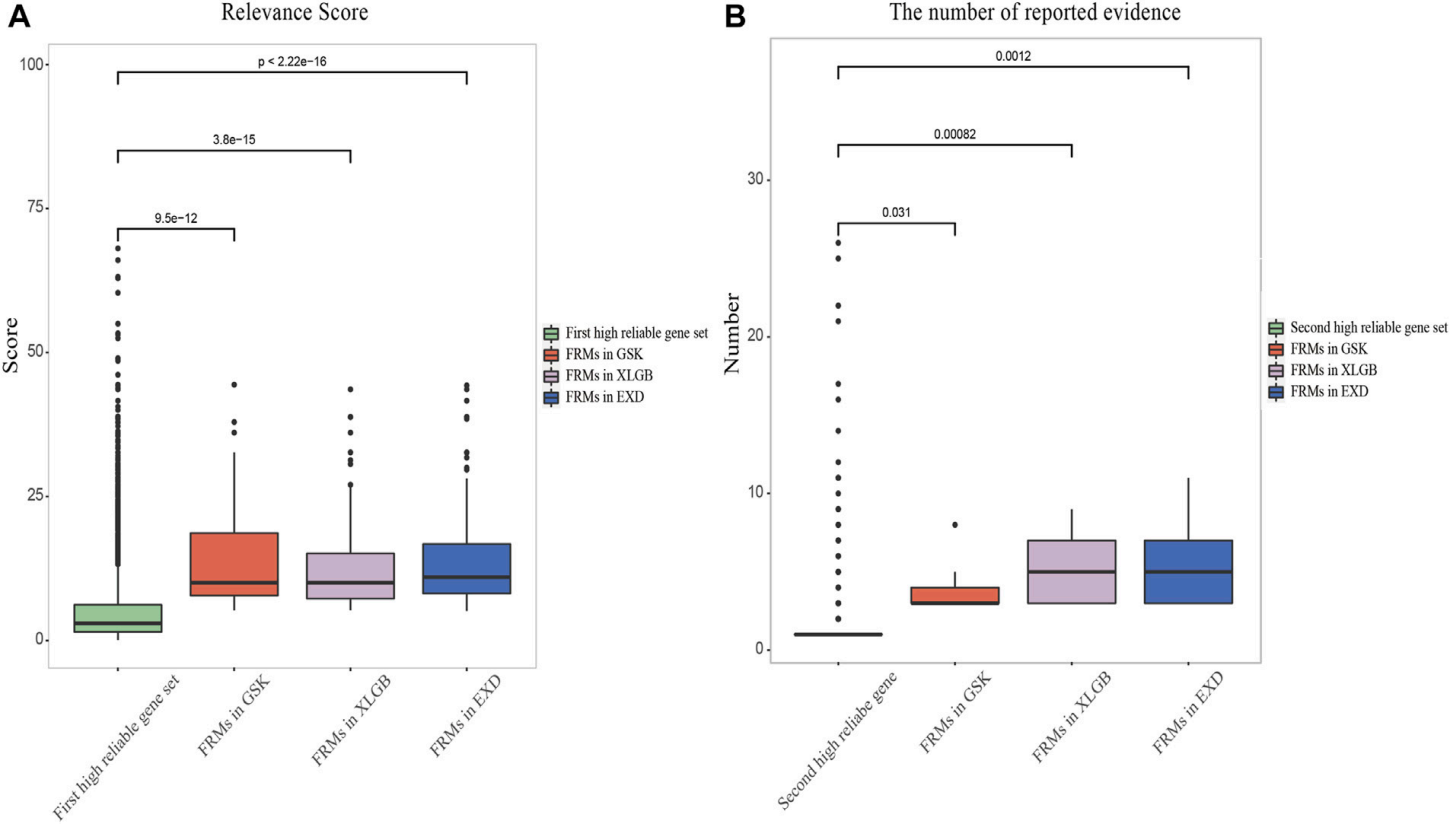
Validation of FRMs. Relevance score **(A)** and the number of reported evidence **(B)** of FRMs and two high reliable pathogenic gene sets of OP were compared.

Herein, we set a criterion for screening two high reliable pathogenic gene sets by calculating the average relevance score and the number of reported evidence of pathogenic genes, respectively. The first high reliable pathogenic gene set was defined as the genes with higher relevance score than the average of all pathogenic genes. The second high reliable pathogenic gene set was defined as the genes with higher number of reported evidence than the average of all pathogenic genes. According to this criterion, the average relevance score and the number of reported evidence of pathogenic genes of OP collected from the database are 5.13 and 2.7, respectively. Thus, in the first high reliable pathogenic gene set, the relevance score of each gene was higher than 5.13. While in the second high reliable pathogenic gene set, the number of reported evidence of each gene was higher than 2.7. The average relevance scores of FRMs in GSK, XLGB, and EXD were 14.22, 12.98, and 14.43, respectively, while the average numbers of reported evidence were 10.5, 7.75, and 8.17, respectively, which were significantly higher than the two high reliable pathogenic gene sets, respectively (Figure 6). The high relevance score and the number of reported evidence can represent a higher degree of association between genes and disease, and the average score and the number of reported evidence of predicted FRMs in three prescriptions were significantly higher than those of the two high reliable pathogenic genes sets. Results showed that the FRMs prediction model could accurately screen genes with higher disease correlation.

#### Validating FRMs Based on Gene Enrichment Pathways Analysis

**FIGURE 7:**
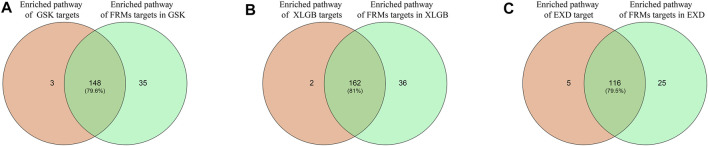
Validation of FRMs. The intersection proportion of FRMs gene enrichment pathways and C-T network gene enrichment pathways in GSK**(A)**, XLGB**(B)**, and EXD**(C)**.

**FIGURE 8:**
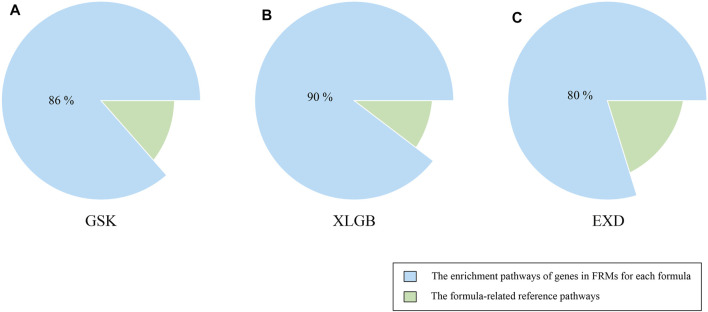
Validation of FRMs. The proportion of the enrichment pathways of genes in FRMs for GSK **(A)**, XLGB **(B)**, and EXD **(C)** to corresponding reference pathways. The formula-related reference pathways were defined as selecting the intersection of the target gene and pathogenic gene enriched pathways.

Another criterion for evaluating the accuracy and reliability of FRMs is to evaluate their functional consistency. According to this method, we used the KEGG enrichment analysis to determine whether the gene enrichment pathways in FRMs could cover the gene enrichment pathways in the C-T network as much as possible. The high pathways coverage indicates that FRMs can represent the complete C-T network at the functional level. Our analysis showed that GSK, XLGB, and EXD FRMs gene enrichment pathways accounted for 79.6, 81, and 79.5% of GSK, XLGB, and EXD complete C-T network gene enrichment pathways, respectively (Figure 7). The results showed that the predicted FRMs can represent the complete C-T network at the functional level, and it also indicated that the predicted FRMs can preserve the functional pathway of herbal medicine prescriptions to the maximum extent. Additionally, to examine whether the FRMs in each prescription have the similar therapeutic function as the original prescription, we defined the prescription-related reference pathways by selecting the intersection of target gene and pathogenic gene enriched pathways. After that, we compared the enrichment pathways of genes in FRMs for each prescription to corresponding reference pathways. The results showed that the proportion of GSK, XLGB, and EXD was 86.47, 89.63, and 79.8%, respectively (Figure 8). It showed that screened FRMs can retain the functional information of the original prescriptions and the intervention function of the original prescriptions to the maximum extent.

### Potential Mechanism Analysis

**FIGURE 9:**
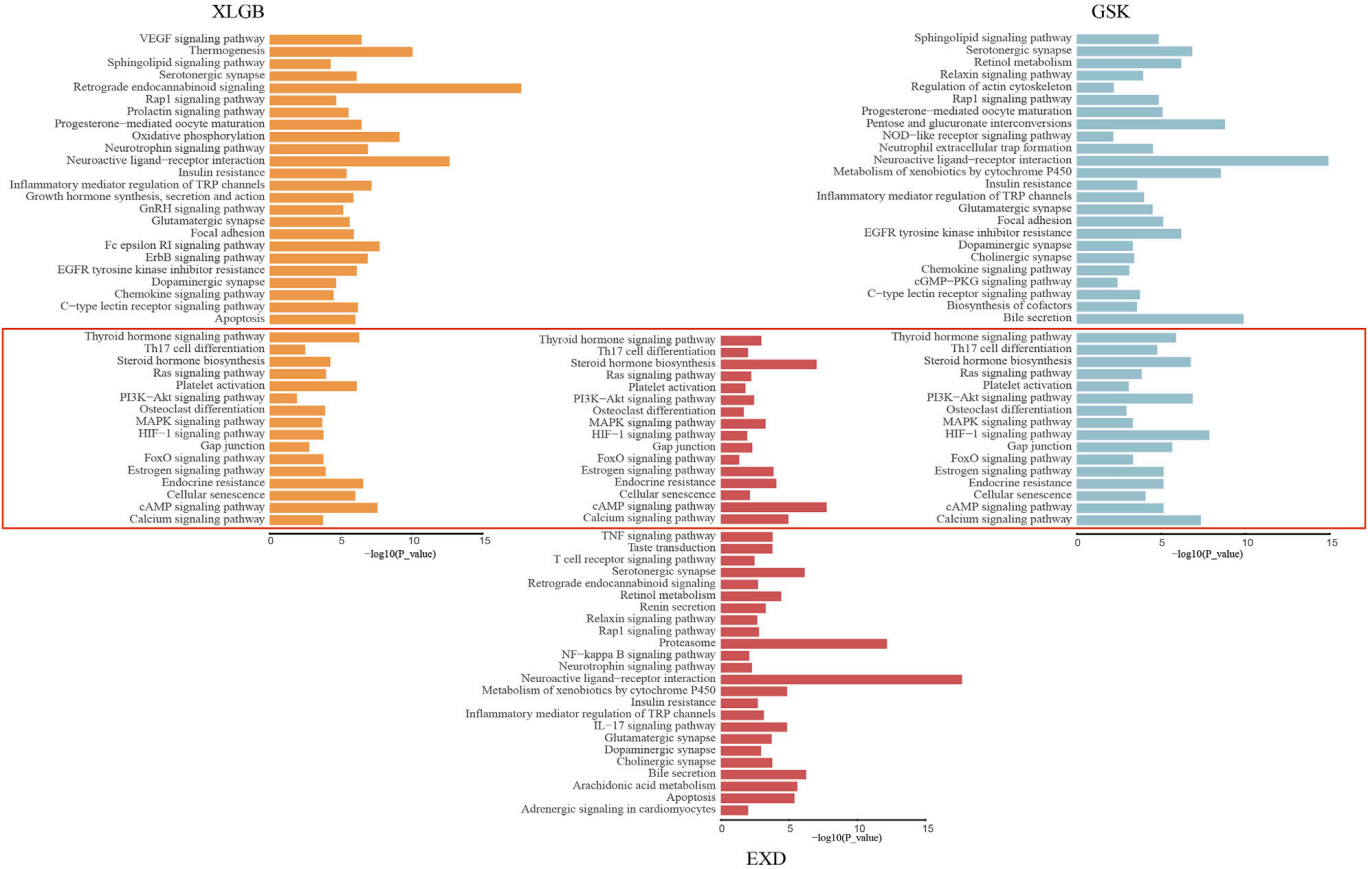
The specific and the common enrichment pathway of FRMs in GSK, XLGB, and EXD.

In order to reveal the potential action mode underlying the treatment of OP by different prescriptions, we performed a pathway enrichment analysis of FRMs genes. Other disease, virus-related, and drug-resistant pathways were removed, and pathways with a count number greater than 12 were retained for further analysis. We found that 40 pathways were enriched in GSK, 66 pathways in XLGB, and 48 pathways in EXD. After combination, the three prescriptions were found to be enriched in 16 identical pathways (Figure 9), for example, steroid hormone biosynthesis (hsa00140), osteoclast differentiation (hsa04380), calcium signaling pathway (hsa04020), MAPK signaling pathway (hsa04010), and PI3K-Akt signaling pathway (hsa04151). Through PubMed literature retrieval, we found that, among these common pathways, osteoclast differentiation, calcium signaling pathway, MAPK signaling pathway, and PI3K-Akt signaling pathway were most reported. Previous studies have shown that the suppression of RANKL-induced calcium signaling inhibits OP in oophorectomy (OVX) mouse models (Chen et al., 2020). MAPK pathway is a major signaling pathway regulating OP. By restraining the production of RANKL-induced ROS and increasing the expression of antioxidant enzymes, intracellular ROS levels are inhibited and the activation of the MAPK pathway is weakened, resulting in the attenuation of downstream proteins, which contributes to the reduction of OP (Yao et al., 2018; Chen et al., 2019). Meanwhile, the PI3K/AKT signaling pathway has been proven to be essential for all stages of bone maturation, differentiation, and bone growth. Inhabiting the PI3K/AKT signaling pathway not only injures the differentiation of chondrocytes but also inhibits the growth of longitudinal bones (Ye et al., 2019).

**FIGURE 10:**
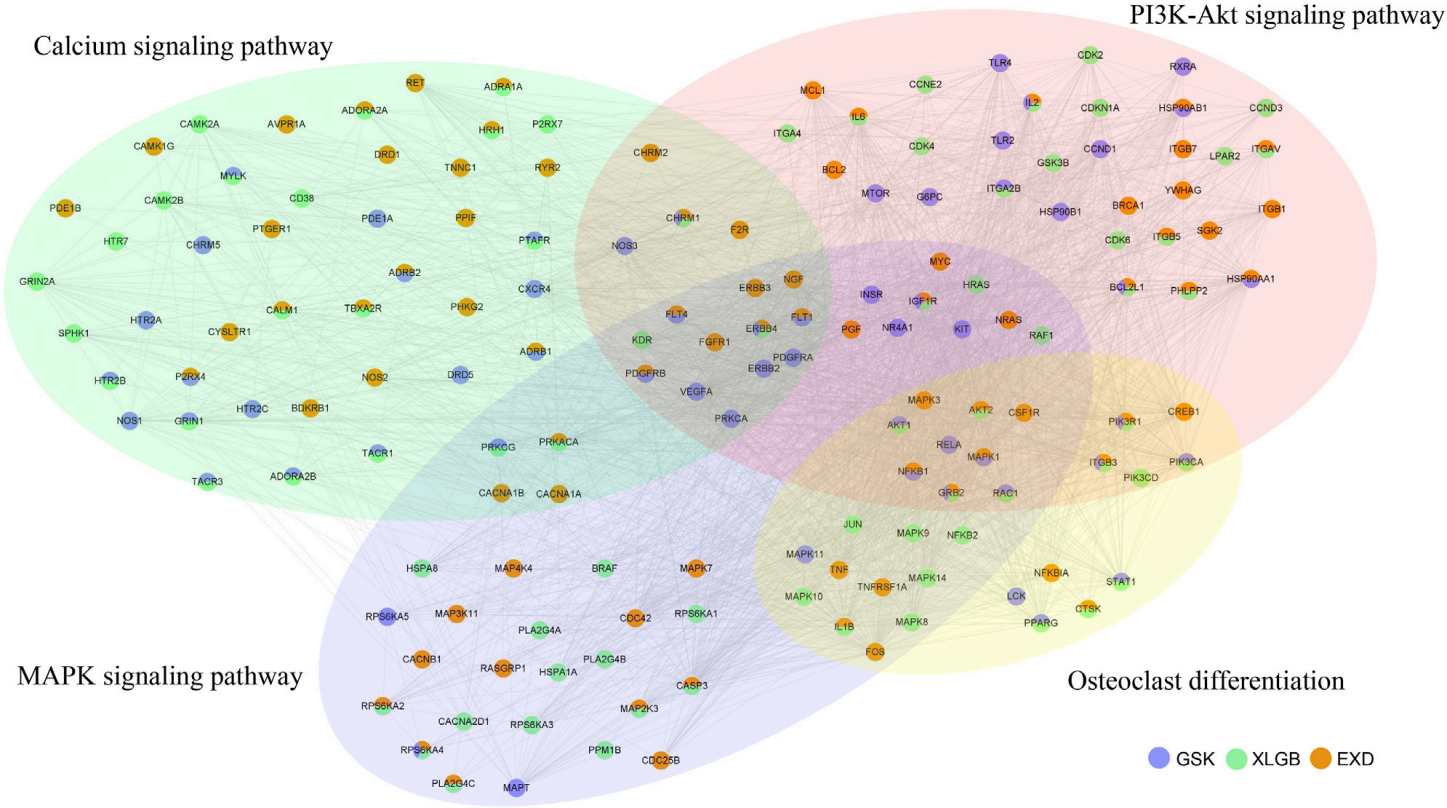
Gene network in the osteoclast differentiation, calcium signaling pathway, MAPK signaling pathway, and PI3K-Akt signaling pathway. Blue nodes represent targets in GSK. Green nodes represent targets in XLGB. Orange nodes represent targets in EXD. Nodes with three colors indicate that the gene is shared by three prescriptions. Nodes in light yellow, light green, light blue, and light red circles represent genes of osteoclast differentiation, calcium signaling pathway, MAPK signaling pathway, and PI3K-Akt signaling pathway, respectively.

Next, osteoclast differentiation, calcium signaling pathway, MAPK signaling pathway, and PI3K-Akt signaling pathway were combined into a gene network (Figure 10). Our analysis found that, in this gene network, GSK, XLGB, and EXD had different and co-functional effects in treating OP. There were 10 genes common to GSK, XLGB, and EXD (ITGB3, IGF1R, CHRM1, GRB2, IL2, BCL2L1, ERBB4, PIK3R1, RPS6KA4, and TACR2). For example, GSK, XLGB, and EXD all have target gene ERBB4, which played a role in OP through PI3K-Akt signaling pathway, MAPK signaling pathway, and calcium signaling pathway, and common gene IL2, which only affected OP through the PI3K-Akt signaling pathway. In the gene network, 27, 37, and 44 genes were unique to GSK, XLGB, and EXD. For example, among the 27 specific genes of GSK, LCK affected OP through osteoclast differentiation; VEGFA played a role in OP through the PI3K-Akt signaling pathway, MAPK signaling pathway, and calcium signaling pathway. Among the 37 unique genes of XLGB, HRH2 simply affected OP through the calcium signaling pathway; JUN impacted OP through the MAPK signaling pathway and osteoclast differentiation pathway. Among the 44 distinct genes of EXD, CACNB1 merely affected OP through the MAPK signaling pathway; CHRM2 influenced OP through the PI3K-Akt signaling pathway and calcium signaling pathway (Figure 10). ITGB3 belongs to the integrin family and is a membrane receptor composed of a subunit and ß subunit, which is involved in cell cycle, cytoskeletal tissue, osteoblast differentiation, and proliferation (Rapisarda et al., 2017; Lopes et al., 2019). ITGB3 can also interact with IL1RN to activate ß-catenin signaling and regulate osteoblast differentiation (Zou et al., 2021). In addition, IGF1R signaling plays an important role in osteoblast-mediated bone formation and promotes osteoblasts differentiation and maturation. During the differentiation of bone marrow stromal cells, IGF1R signaling pathway is controlled by PI3K/Akt and inhibits osteoblast apoptosis (Fang et al., 2019).

Among the specific genes of GSK, cyclin D1 (CCND1) is a member of the protein kinases family associated with cell cycle regulation (Guo et al., 2013). In addition, CCND1 is often overexpressed in human diseases through translocation, amplification, or post-transcriptional regulation (Xu and Lin, 2018). Studies have found that controlling the expression of CCND1 can regulate the proliferation, differentiation, mineralization, and apoptosis of osteoblasts, therefore treating OP (Wang and Cai, 2020). In addition, it was shown that chemokine receptor-4 (CXCR4) overexpression in mesenchymal stem cells can promote BMD increase, improve MSC migration to bones, enhance MSC effects, and prevent bone loss in OVX mice after oophorectomy (Cho et al., 2009).

Among the specific genes of XLGB, CD38 has been shown to play a role in bone remodeling. It is expressed in osteoclasts, and when activated, it increases IL-6 release and inhibits bone resorption (Sun et al., 1999). Meanwhile, osteoblasts and osteoclasts express P2X7R, which regulates differentiation, function, and longevity of both cell types (Agrawal and Gartland, 2015). In the mouse model of postmenopausal osteoporosis, P2X7 has been shown to hinder bone loss. After the deletion of P2X7R in ovariectomized mice, significant bone loss, osteoblast reduction, and osteoclast increase were observed. Under normal physiological conditions, the loss of P2X7R leads to osteopenic-like bone phenotype (Wang et al., 2018b).

Among the specific genes of EXD, BCL2 is a gene that inhibits apoptosis. Studies have shown that upregulation of BCL2 may inhibit bone loss, while upregulation of BCL2 in osteoblasts can restrain osteoblast differentiation and cause osteocyte apoptosis (Moriishi et al., 2011). Colony stimulating factor 1 receptor (CSF-1R or c-FMS) is a tyrosine kinase receptor, a receptor for CSF-1, which is delivered by osteoblasts and promotes the proliferation of osteoclast progenitor cells through the combination of CSF-1R and receptor activator of nuclear factor-kappaB ligand (RANKL), leading to the formation of mature osteoclasts (Wittrant et al., 2009). Previous studies have shown that the CSF-1/CSF-1R signaling pathway affects the expression of RANK in osteoclasts and RANKL binds to RANK to induce the cascade of MAPKs, PI3K, and NF-κB signaling and finally generates NFATc1, which is the major regulator of osteoclast differentiation. The inhibition of CSF-1R on osteoclasts hinders the proliferation, differentiation, and survival of osteoclasts and downregulates the formation of osteoclast markers (TRAP) (Zinnia and Khademul Islam, 2021).

The above results indicate that the FRMs of different prescriptions have different and co-functional effects in treating OP. They provide a possible mechanism reference for us to reveal multiple treatments for the same disease.

### Screen Key Component Groups

Osteoclast differentiation, calcium signaling pathway, PI3K-Akt signaling pathway, and MAPK signaling pathway were combined into an integrated pathway. A novel component importance calculation method that combined the information gain and target influence was designed and employed to screen the key component groups in different prescriptions. The components with a Q score of more than 0.01 were defined as potential important components in each prescription. Based on this method, we obtained 24, 23, and 22 potential important components in GSK, XLGB, and EXD, respectively. To prove the effectiveness of each potential important component screened by the model, we selected the highest Q score component in each of the three prescriptions and randomly selected one, one, and two components from GSK, XLGB, and EXD, respectively. Finally, we selected quercetin, isoliquiritigenin, rutaecarpine, isofraxidin, and secoisolariciresinol for further validation. The effectiveness of these components was further verified *in vitro* to prove the model’s reliability (Table 3).

**TABLE 3:** The potential important components and score in GSK, XLGB, and EXD.

Formula	Name	Component	Q
GSK	Phenylalanine	GSK-430	1
GSK	Quercetin	GSK-136	0.529699172
GSK	Secoisolariciresinol	GSK-264	0.468361473
GSK	Apigenin 7,4′-dimethyl ether	GSK-51	0.349024322
GSK	Arginine	GSK-323	0.264370699
GSK	Tyrosine	GSK-435	0.211996592
GSK	Pinoresinol	GSK-263	0.21119811
GSK	Xanthogalenol	GSK-317	0.157596558
GSK	Gamma-aminobutyric acid	GSK-350	0.157417777
GSK	Isoolivil	GSK-26	0.148203935
GSK	Iriflophenone	GSK-310	0.147059487
GSK	Methionine	GSK-487	0.1389871
GSK	Artonin U	GSK-88	0.135082763
GSK	Lariciresinol	GSK-376	0.127357247
GSK	Histidine	GSK-439	0.120541692
GSK	1,2-Bis(4-hydroxy-3-methoxyphenyl)propane-1,3-diol	GSK-138	0.117705025
GSK	(2S,3S,4R,5S,6R)-2-(Hydroxymethyl)-6-[2-(4-hydroxyphenyl)ethoxy]oxane-3,4,5-triol	GSK-47	0.11770502
GSK	3,4-Dihydroxybenzaldehyde	GSK-462	0.115043307
GSK	Vladinol F	GSK-133	0.114306628
GSK	1-Hexanol	GSK-24	0.113316944
GSK	Apigenin	GSK-2	0.112568974
GSK	Methyleugenol	GSK-10	0.107187098
GSK	Methyl rosmarinate	GSK-568	0.105064369
GSK	Tanshinone IIA	GSK-606	0.100684748
XLGB	Phenylalanine	XLGB-174	1
XLGB	Tyrosine	XLGB-187	0.265722595
XLGB	Quercetin	XLGB-35	0.419289486
XLGB	Nicotinamide	XLGB-516	0.237387556
XLGB	Methionine	XLGB-337	0.161318712
XLGB	Lysine	XLGB-186	0.122755389
XLGB	Japonine	XLGB-498	0.143756394
XLGB	Isoliquiritigenin	XLGB-66	0.105348726
XLGB	Huazhongilexin	XLGB-147	0.172357865
XLGB	Histidine	XLGB-176	0.145081365
XLGB	Glycine	XLGB-165	0.181153762
XLGB	Ethyl beta-d-galactopyranoside	XLGB-164	0.179295726
XLGB	Chrysoeriol	XLGB-82	0.139191528
XLGB	Artonin U	XLGB-120	0.207032871
XLGB	Arginine	XLGB-189	0.314224575
XLGB	Apigenin 7,4′-dimethyl ether	XLGB-83	0.251858762
XLGB	Apigenin	XLGB-34	0.164693641
XLGB	3-Hexenyl-beta-glucopyranoside	XLGB-114	0.103747239
XLGB	3,4-Dihydroxybenzaldehyde	XLGB-231	0.158023093
XLGB	2-Decenal	XLGB-37	0.12526283
XLGB	2,6,4′-Trihydroxy-4-methoxybenzophenone	XLGB-510	0.267516823
XLGB	1,2-Bis(4-hydroxy-3-methoxyphenyl)propane-1,3-diol	XLGB-8	0.376297348
XLGB	1-(3,4-Dihydroxyphenyl)-2-hydroxyethanone	XLGB-350	0.118592977
EXD	Isoolivil	EXD-117	1
EXD	Vanillyl alcohol	EXD-616	0.125075771
EXD	Tyrosine	EXD-421	0.193500377
EXD	Rutaecarpine	EXD-631	0.114443255
EXD	Quercetin	EXD-226	0.350841822
EXD	Phenylalanine	EXD-400	0.204988715
EXD	Nicotinamide	EXD-751	0.107475835
EXD	N-cis-Feruloyltyramine	EXD-674	0.113378648
EXD	Methyl nonyl ketone	EXD-454	0.157576077
EXD	Methionine	EXD-316	0.262915243
EXD	Isoliquiritigenin	EXD-125	0.570518314
EXD	Isofraxidin	EXD-495	0.145679135
EXD	Dibutyl phthalate	EXD-443	0.159583501
EXD	Coniferyl ferulate	EXD-367	0.224742828
EXD	Asperglaucide	EXD-679	0.110398041
EXD	Arginine	EXD-424	0.17722632
EXD	Apigenin 7,4′-dimethyl ether	EXD-141	0.522823729
EXD	2-Phenylethanol	EXD-456	0.145887101
EXD	2-Carbomethoxy-9,10-anthraquinone	EXD-526	0.126521782
EXD	2,6,4′-Trihydroxy-4-methoxybenzophenone	EXD-745	0.108038542
EXD	1-Hydroxy-3-methoxyanthracene-9,10-dione	EXD-517	0.12688071
EXD	1,3-Dimethoxybenzene	EXD-9	0.100024253

### Experimental Validation *In Vitro*

**FIGURE 11:**
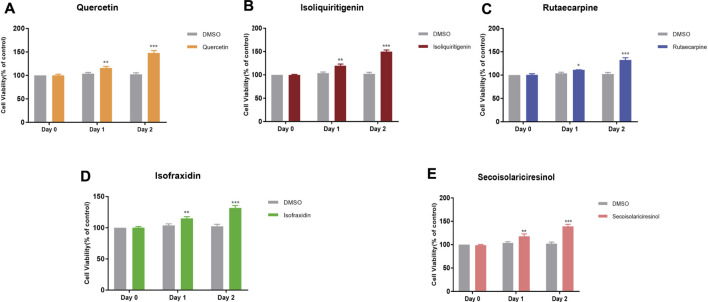
Effects of quercetin **(A)**, isoliquiritigenin **(B)**, rutaecarpine **(C)**, isofraxidin **(D)**, and secoisolariciresinol **(E)** on cell viabilities on MC3T3-E1 cells. *p < 0.05, **p < 0.01, ***p < 0.001 compared with DMSO group.

MC3T3-E1 is an osteoblast strain constructed from C57BL/6 mouse cranial parietal cells, which has biological characteristics of osteoblasts, such as ALP active type Ⅰ collagen synthesis and matrix calcification, and is often used as a cellular model for bone metabolism studies. In order to assess the reliability of our model, the CCK8 method was used to validate the effects of the key components (quercetin, isoliquiritigenin, rutaecarpine, isofraxidin, and secoisolariciresinol) on the MC3T3-E1 cells at different time points (Day 0, Day 1, Day 2). The results showed that the cell viabilities of MC3T3-E1 cells were 115.93, 119.74, 111.27, 115.11, and 117.02% after exposure to 5 μM quercetin, isoliquiritigenin, rutaecarpine, isofraxidin, and secoisolariciresinol on Day 1, respectively (Figure 11). On Day 2, the cell viabilities of five components were 147.88, 149.88, 132.34, 131.87, and 133.02%, respectively (Figure 11). Compared with the control group (DMSO), the cell viability of the MC3T3-E1 cells markedly increased after treatment with five components at Day 1 and Day 2. Our results suggested that quercetin, isoliquiritigenin, rutaecarpine, isofraxidin, and secoisolariciresinol could increase the viabilities of MC3T3-E1 cells.

## Discussion

OP causes fractures and other complications, which increase the physical pain of patients and bring great pressure and burden to the society and family. For the treatments of OP, drugs that promote bone mineralization, inhibit bone resorption, and promote bone formation are widely used (Das and Crockett, 2013). However, these drugs are expensive and have side effects. Thus, it is desirable to choose an alternative medicine to address this issue. Herbal medicine prescriptions have significant advantages in the treatment of OP because of their remarkable efficacy and few side effects. However, due to the characteristic of “multi-components-multi-targets-multi-pathways,” their mechanism of action is not yet clear. Therefore, it is required to exploit new methods to study the pharmacological material basis and molecular mechanism of herbal medicine prescriptions and make a scientific interpretation.

Herbal informatics is an interdisciplinary subject that integrates Chinese medicine, computer science, biology, mathematics, multi-directional pharmacology, and other disciplines. It researches complex herbal medicine systems by systematically observing the intervention and influence of drugs on disease networks (Guo et al., 2021). Herbal informatics has been widely used in herbal medicine. For example, Wang Kexin et al. systematically interpreted the compatibility of Huanglian Jiedu Decoction in treating diseases (Wang et al., 2020b). Gao Yao et al. used the method of herbal informatics to analyze the mechanism of LCW in treating Systemic Lupus Erythematosus (Gao et al., 2020). Yang Lang et al. designed a new network pharmacology method to decode the mechanism of EXD in treating OP (Yang et al., 2021). These studies demonstrate the reliability of herbal informatics.

In this study, after ADME screening, 517 active components were obtained. Among the three prescriptions, there were 88 common components; there were 65, 37, and 232 specific components in GSK, XLGB, and EXD, respectively. This suggests that the three prescriptions impact OP treatment through common and specific components.

To quickly extract vital information from the complex C-T networks, we designed a novel function motif discovery model and a genetic-based optimization model and obtained 11, 13, and 15 FRMs (*p* < 0.05). The FRMs were used to detect the latent action mode of GSK, XLGB, and EXD in the clinical therapeutics of OP.

The function motif discovery model based on a random walk adopts the idea of double-layer coding, which can greatly simplify the coding length. Nodes are divided into N categories with different numbers, and the same coding is used in each category so that the coding length can be saved. The genetic-based optimization model has good global search ability and can quickly search for all solutions in the solution space without falling into the trap of fast descent of local optimal solutions. Moreover, it can conveniently carry out distributed calculations, accelerate the solving speed, and have strong convergence using its inherent parallelism. At the same time, searching from the group has the potential to be parallel. It can compare multiple individuals at the same time and use the evaluation function to evaluate individuals. The process is simple. In the iterative process, the probability mechanism is used to iterate, and the selection of individuals is random to avoid accidental results. The genetic algorithm (GA) can retain good individuals and maintain the group’s diversity. The information gain inspects the feature’s contribution to the whole system and considers both the occurrence and nonoccurrence of the feature. Meanwhile, it considers comprehensively and uses the statistical attributes of all samples to reduce the sensitivity to noise.

The accuracy and reliability of FRMs were evaluated using the relevance score, the number of reported evidence, and the coverage of functional pathways of FRMs. Results showed that the predicted FRMs overlapped with the C-T network in pathogenic genes and functional pathways at high coverage.

Next, we conducted pathway enrichment analysis for each FRMs and found that 16 enriched pathways were shared by three prescriptions. Through a literature search, we found that osteoclast differentiation, calcium signaling pathway, MAPK signaling pathway, and PI3K-Akt signaling pathway were most reported. Recent research has shown that RANKL-induced ROS signaling regulates the MAPK and NF-κB activity, and the loss of NF-κB signaling in mouse models causes bone defect formation, leading to osteoporosis (Iotsova et al., 1997). RANKL can stimulate the three main members of the MAPK signaling pathway, JNK, ERK, and P38, therefore impacting the differentiation of osteoclasts (Boyle et al., 2003). In the OVX mouse model, the reduction of ROS level and the inhibition of the MAPK signaling pathway can inhibit osteoclast differentiation and reduce bone loss (Xiao et al., 2020). PI3K/AKT signaling pathway exists in mammalian cells; is involved in cell proliferation, metastasis, and apoptosis; and regulates the functions of osteoblasts and osteoclasts by affecting their formation, proliferation, differentiation, and apoptosis (Agas et al., 2013) (Qu et al., 2014). Activating the PI3K/AKT signaling pathway affects the upregulation of the expression of osteogenic differentiation marker genes, thereby promoting the proliferation and differentiation of osteoblasts (Lu et al., 2017). Inhibition of the PI3K/AKT signaling pathway can reduce the bone resorption ability of osteoclasts (Zhang et al., 2020), thus inhibiting the occurrence of osteoporosis.

Furthermore, we found that some enriched genes were shared by four pathways, but others were specific to one pathway, and these genes all played different roles in the influence of OP, indicating that the FRMs of different prescriptions have different and co-functional effects in the treatment of OP. It provides a possible mechanism reference for us to reveal multiple treatments for the same disease.

Then, we devised a novel component importance calculation method that combined the information gain and target influence to screen the key component groups in different prescriptions. The components with a Q score more than 0.01 were defined as potential important components in each prescription. Using this new method, we obtained 24, 23, and 22 potential important components in GSK, XLGB, and EXD; selected the highest important component; and randomly selected components as the key component groups of the three prescriptions, including quercetin, isoliquiritigenin, rutaecarpine, isofraxidin, and secoisolariciresinol. The effectiveness of these components was verified by *in vitro* experiments to prove the model’s reliability.

*In vitro* cell experiments also showed that these components can increase the viability of MC3T3-E1 cells, speculating that these components have a certain role in treating OP. Additionally, to better evaluate the dependability of our proposed network pharmacological model, we will conduct *in vivo* studies in future studies.

However, there are still some limitations in this study. First, more ingredients from key component groups should be selected for validating the reliability of our method and model. Second, the action mode of GSK, XLGB, and EXD in the clinic therapy of OP is not verified in this study. Meanwhile, the issue of drug dose should be considered in future studies.

In conclusion, we propose a new network pharmacology strategy that reveals the hidden mechanisms of different prescriptions for OP through new bioinformatics models and experimental validation, providing a new web-based approach for herbal medicine treatment of complex diseases.

## Data Availability

The original contributions presented in the study are included in the article/Supplementary Material, further inquiries can be directed to the corresponding authors.

## Abbreviations

OP, osteoporosis; GSK, Gushukang Granules; XLGB, Xianling Gubao Capsules; EXD, Er-xian Decoction; SDFM, Significant Different Functional Modes; FRMs, FRM, Functional Response Motif; C-T network, component-target network.
