# Supplementary figures and images for: Uncovering Hidden Mechanisms of Different Prescriptions Treatment for Osteoporosis via Novel Bioinformatics Model and Experiment Validation
Source: Front Cell Dev Biol. 2022 Feb 8;10:831894. doi: 10.3389/fcell.2022.831894 (PMC8861325; doi:10.3389/fcell.2022.831894)

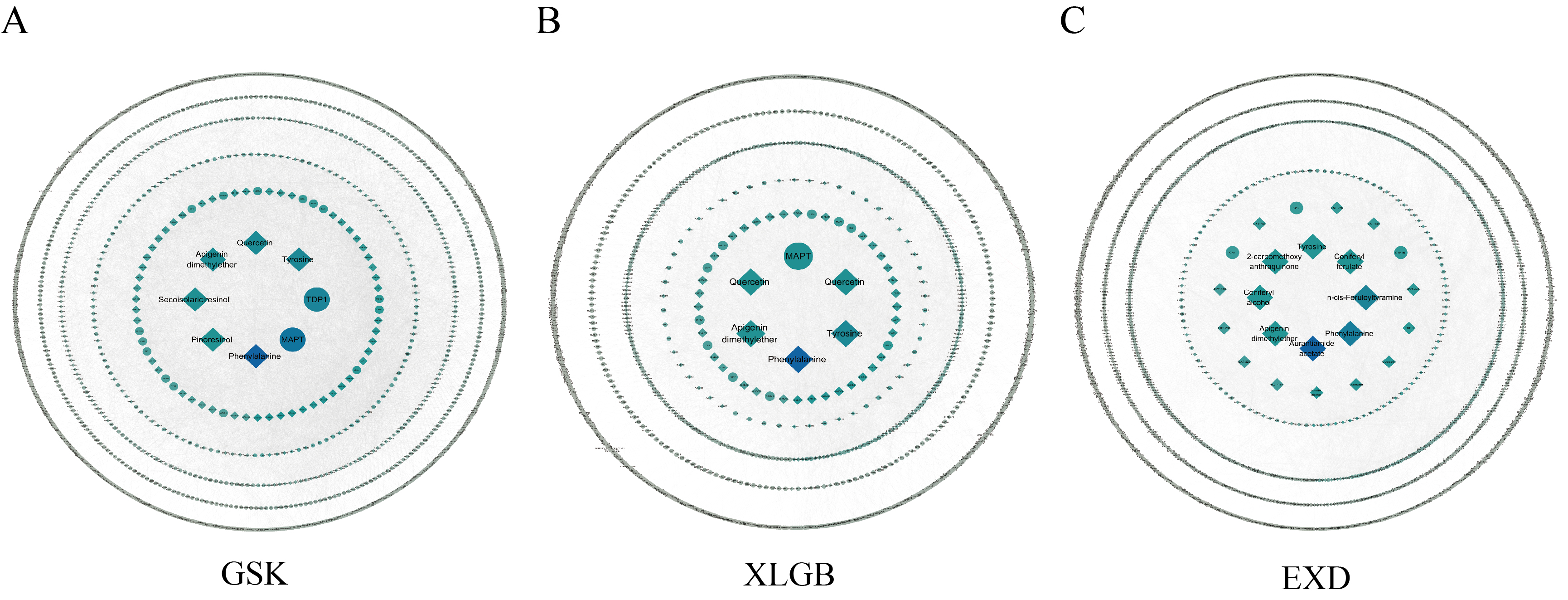

Supplement: Supplementary file 2 [file Image1.JPEG]
