# Supplementary material for: Uncovering Hidden Mechanisms of Different Prescriptions Treatment for Osteoporosis via Novel Bioinformatics Model and Experiment Validation
Source: Front Cell Dev Biol. 2022 Feb 8;10:831894. doi: 10.3389/fcell.2022.831894 (PMC8861325; doi:10.3389/fcell.2022.831894)
Supplement: Supplementary file 4 [file DataSheet1.docx]

Supplementary Material

# Supplementary Figures and Tables

Supplementary material related to this article can be found, in the online version.

## Supplementary Figures

**
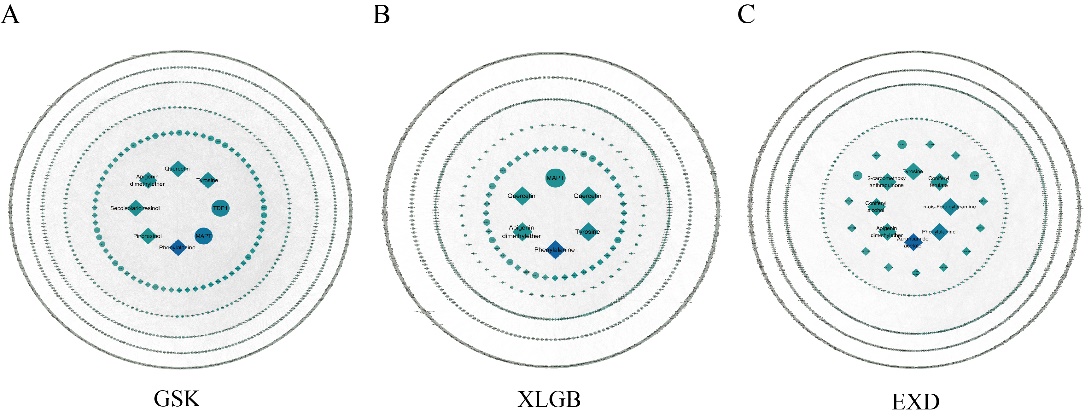
**

**Supplementary Figure 1.** The C-T network of GSK (A), XLGB (B), and EXD (C). The rhombic nodes present the components in GSK, XLGB, and EXD. The circular nodes show the targets in GSK, XLGB, and EXD. The depth of the color represents the degree of the node. The higher degree of the node, the darker the color is.

**Supplementary TABLE 1.** The detailed information of components in GSK, XLGB, and EXD.

**Supplementary TABLE 2.** The detailed information of components after SwissADME screening in GSK, XLGB, and EXD.
